# Genetic diversity, population structure, and selection of breeder germplasm subsets from the USDA sweetpotato (*Ipomoea batatas*) collection

**DOI:** 10.3389/fpls.2022.1022555

**Published:** 2023-02-02

**Authors:** Tyler J. Slonecki, William B. Rutter, Bode A. Olukolu, G. Craig Yencho, D. Michael Jackson, Phillip A. Wadl

**Affiliations:** ^1^ United States Vegetable Laboratory, Agricultural Research Service, United States Department of Agriculture, Charleston, SC, United States; ^2^ Department of Entomology and Plant Pathology, University of Tennessee, Knoxville, TN, United States; ^3^ Department of Horticultural Science, North Carolina State University, Raleigh, NC, United States

**Keywords:** sweetpotato, convolvulaceae, USDA germplasm, SNPs, DAPC, breeder subsets, genotyping-by-sequencing (GBS), polyploid

## Abstract

Sweetpotato (*Ipomoea batatas*) is the sixth most important food crop and plays a critical role in maintaining food security worldwide. Support for sweetpotato improvement research in breeding and genetics programs, and maintenance of sweetpotato germplasm collections is essential for preserving food security for future generations. Germplasm collections seek to preserve phenotypic and genotypic diversity through accession characterization. However, due to its genetic complexity, high heterogeneity, polyploid genome, phenotypic plasticity, and high flower production variability, sweetpotato genetic characterization is challenging. Here, we characterize the genetic diversity and population structure of 604 accessions from the sweetpotato germplasm collection maintained by the United States Department of Agriculture (USDA), Agricultural Research Service (ARS), Plant Genetic Resources Conservation Unit (PGRCU) in Griffin, Georgia, United States. Using the genotyping-by-sequencing platform (GBSpoly) and bioinformatic pipelines (ngsComposer and GBSapp), a total of 102,870 polymorphic SNPs with hexaploid dosage calls were identified from the 604 accessions. Discriminant analysis of principal components (DAPC) and Bayesian clustering identified six unique genetic groupings across seven broad geographic regions. Genetic diversity analyses using the hexaploid data set revealed ample genetic diversity among the analyzed collection in concordance with previous analyses. Following population structure and diversity analyses, breeder germplasm subsets of 24, 48, 96, and 384 accessions were established using K-means clustering with manual selection to maintain phenotypic and genotypic diversity. The genetic characterization of the PGRCU sweetpotato germplasm collection and breeder germplasm subsets developed in this study provide the foundation for future association studies and serve as precursors toward phenotyping studies aimed at linking genotype with phenotype.

## Introduction

Sweetpotato (*Ipomoea batatas*) plays an important role in maintaining food security across the world. As of 2018, it was the sixth most important food crop in the world and has been rising in popularity in the United States since 2000 (Economic Research Service, United States Department Of Agriculture, 2016). It serves as a staple source of carbohydrates, energy, and phytonutrients for human consumption and animal feeding (Mohanraj and Sivasankar, 2014). More than 8 million hectares of land are used to produce 89 million tons of sweetpotato worldwide. Currently, the largest producer of sweetpotato is China, while the United States is within the top 10 producers (United Nations, 2020). North Carolina, the largest producer of sweetpotato in the United States, harvested 1.6 billion pounds in 2015 (Economic Research Service, United States Department Of Agriculture, 2016). Its rise in popularity in the United States is likely attributed to public awareness of sweetpotato nutritional and macronutrient benefits (it provides over 90% of nutrients per calorie required for most people) (Mohanraj and Sivasankar, 2014), the introduction of the sweetpotato french-fry and many other value-added products, and a public push toward healthier diets and lifestyles. Primarily due to its lower glycemic index and diverse nutritional profile, sweetpotato is viewed as a healthier source of carbohydrates compared to potato and may be particularly beneficial for people with diabetes, obesity, or cardiovascular disease (Wright et al., 2017; Oloniyo et al., 2021). Furthermore, sweetpotato is a good source of vitamins (A, B1, B2, B3, B6, C, E, biotin and pantothenic acid), minerals (iron, calcium, magnesium, manganese, and potassium), and dietary fiber and may be an affordable source to combat nutritional deficiencies in developing countries (Mohanraj and Sivasankar, 2014; Wang et al., 2016).

The modern cultivated sweetpotato genome is a hexaploid (2n = 6x = 90), however a few reports have observed tetraploid (2n = 4x = 60) sweetpotato (Bohac et al., 1993; Roullier et al., 2013). While the exact origins of the cultivated sweetpotato remain a mystery, there are two major competing proposed explanations. The first hypothesis suggests that there was a two-step polyploidization event stemming from a single progenitor (*I. trifida*) based on an initial autopolyploidization that produced a tetraploid, followed by a tetraploid-diploid cross. The second hypothesis follows the same initial autopolyploidization of a species followed by tetraploid-diploid hybridization with a different species (likely *I. trifida* and *I. batatas*) (Austin, 1988, Oracion et al., 1990; Roullier et al., 2013; Yang et al., 2017; Muñoz-Rodríguez et al., 2018). Recently, Mollinari et al. proposed a hexasomic-bivalent inheritance model that may help elucidate sweetpotato origins and allow for efficient application of molecular techniques (Mollinari et al., 2020) and Muñoz-Rodríguez et al. identified a new tetraploid species, *Ipomoea aequatoriensis*, that may be sweetpotato’s closest tetraploid relative known to date (Muñoz-Rodríguez et al., 2022). Due to the complexity of polyploid genetics and segregation ratios (Lebot, 2010), sweetpotato can be notoriously difficult to breed. This complexity has contributed to a lag in sweetpotato genotyping and germplasm collection characterization. However, with decreased costs of genotyping and improved bioinformatic tools, genotypic characterization of large germplasm collections has become feasible and more commonplace. In 2018, Gouesnard et al. characterized the genetic diversity of 1191 maize lines using genotyping-by-sequencing (GBS) (Gouesnard et al., 2017). More recently, Milner et al. assessed population structure and domestication of 21,406 barley lines using GBS as well (Milner et al., 2019). Other high-throughput genotyping technologies that can be used to characterize germplasm collection include specific length amplified fragment (SLAF) (Sun et al., 2013), amplified fragment length polymorphism (AFLP) (Kriegner et al., 2003) and Diversity Array Technology (DArT) (Ordidge et al., 2018). Specifically, in sweetpotato SLAF was used to identify QTL associated with color-related traits of purple varieties (Yan et al., 2021) and population structure analysis of a core set of 197 accessions (Su et al., 2017) and AFLP markers have been used to dramatically increase the number of mapper markers and linkage groups in select cultivars (Cervantes-Flores et al., 2008; Xiao et al., 2015; Monden and Tahara, 2017). Furthermore, other resources such as assembled and annotated reference genomes (Isobe et al., 2019; Al-Mohanna et al., 2020) facilitate characterization of diversity and population structure by enabling the ability to map sequences and generate dosage-based variant calls.

One of the primary objectives of a crop genebank is to preserve as much genetic diversity as possible to safeguard genetic resources and ensure future agricultural production with resilient crops. Access to genetic diversity and characterization of crops is a crucial component of breeding programs and complementary research. Sweetpotato genetic improvement and preservation is particularly challenging due to its genetic complexity and polyploid genome (Jones et al., 1986). The crop will continue to benefit from advances in genetic diversity characterization and molecular marker identification tools. Coupled with more efficient phenotype collection, these genomic resources and molecular markers will improve the effectiveness of breeding strategies, particularly for programs that spend substantial resources maintaining clonal viability and genetic integrity. Using these tools, programs can create breeder subsets of distinct individuals that maintain genetic and phenotypic diversity while reducing collection maintenance and freeing up more resources for complementary research. Franco-Duran et al. (2019) proposed the development of breeder subsets for plant breeders or molecular geneticists, rather than the classical core germplasm sets that a genebank manager would be interested in to conserve a fixed proportion of a germplasm collection. These breeder subsets or representative samples from the collection possess characteristics of value and for example may represent a specific geographic region, adaptation, and/or unique responses to abiotic or biotic stresses. Marker-assisted and genomic-assisted breeding and identification of breeder subset is particularly beneficial for sweetpotato programs as long-term field evaluation is costly and time-consuming and backcrossing to introgress simple or oligogenic traits is not easy (Wadl et al., 2018). Furthermore, the sparse passport and characterization data available for the PGRCU sweetpotato collection provides breeders/geneticists with reasonable sized germplasm sets for further characterization.

The accessions analyzed in this study were assembled and made available through the sweetpotato germplasm collection maintained by the United States Department of Agriculture (USDA), Agricultural Research Service (ARS), Plant Genetic Resources Conservation Unit (PGRCU) in Griffin, Georgia, United States. This hexaploid *I. batatas* collection consists of clones obtained in collaboration with several national and international programs and organizations over the course of many decades. This collection supports breeding and genetics programs and serves as the genetic foundation for ongoing sweetpotato improvement research including phenotyping of storage root, foliage, and growth characteristics for over 700 accessions in the PGCRU collection (Jackson et al., 2018; Wadl et al., 2018; Jackson et al., 2019; Jackson et al., 2020). These studies have revealed the abundant phenotypic diversity for root and vegetative phenotypic characteristics in the PGRCU collection; however, information regarding genetic diversity of the collection is lacking.

In this study, we aimed to characterize the genetic diversity and population structure of the PGRCU sweetpotato collection and identify a breeder subset of accessions to allow for more efficient breeding program maintenance and selection in future studies. Determination of the PGRCU genetic diversity, population structure, and breeder subsets, coupled with comprehensive genotyping from this dataset will improve the efficiency and efficacy of the characterization of this germplasm collection and contribute significant genomic resources toward collective sweetpotato knowledge.

## Materials and methods

### Plant materials, DNA isolation, genotyping, and SNP/dosage calling

The sweetpotato accessions analyzed in this study were assembled from the USDA, ARS, PGRCU germplasm repository. Initially, 779 germplasm accessions were genotyped, however 175 were discarded for either not having sufficient quality genotyping data or incorrect phenotype data based on the passport information available on the GRIN-global database. In all, a total of 604 germplasm accessions were used for analyses. The collection consisted of samples from across 7 defined regions (Africa, Caribbean, Central America, Far East, North America, Pacific Islands, and South America) and over 35 different countries (**Table 1** and **Supplementary Table S1**).

**Table 1:** Collection location, breeder subset membership, and genetic diversity indices of 604 sweetpotato (*Ipomoea batatas*) accessions.

Region (no. accessions)	No. accessions in each breeder subset				k	PIC	Ae	Ar	I	NP
Total (n=604)	24	48	96	384	5.503	0.353	2.076	6.226	0.797	5.503
Africa (n=47)	2	3	5	30	3.837	0.338	2.017	4.847	0.731	0.131
Caribbean (n=31)	0	1	4	22	3.844	0.357	2.090	4.967	0.769	0.129
Central America (n=33)	2	4	7	20	3.906	0.359	2.080	4.970	0.775	0.186
Far East (n=104)	5	10	21	80	3.807	0.346	2.080	4.684	0.751	0.061
North America (n=168)	8	12	26	108	3.664	0.321	2.010	4.463	0.699	0.052
Pacific Islands (n=102)	4	12	20	72	3.827	0.345	2.069	4.710	0.750	0.065
South America (n=119)	3	6	13	52	3.577	0.312	1.966	4.431	0.678	0.152

k, number of distinct alleles at a locus; PIC, polymorphic information content; Ae, effective number of alleles, Ar, allele richness; I, Shannon’s information index; NP, number of private alleles.

Complete DNA isolation, genotyping-by-sequencing for heterozygous and polyplopid genomes (GBSpoly), quality filtering of fastq data (ngsComposer: https://github.com/bodeolukolu/ngsComposer (Kuster et al., 2021), and SNP calling, and dosage calling (GBSapp: https://github.com/bodeolukolu/GBSapp) were performed as described previously (Wadl et al., 2018). Briefly, a DNeasy Plant Mini Kit (Qiagen) was used to isolate total genomic DNA from lyophilized leaf tissue. After assessing DNA integrity, purity, and concentration (NanoDrop 2000 spectrophotometer, ThermoFisher), a modified GBS method (GBSpoly) was employed. Isolated genomic DNA was double digested with *Cvi*AII and *Tse*I. Barcode adapter oligos (6-9 bp variable length barcodes downstream of 6-bp buffer sequences) were ligated to digested fragments, while destroying the restriction recognition site. Pooled aliquots were double digested again (*Cvi*AII and *Tse*I) to eliminate chimeric sequence ligations. Fragments were purified again and filtered for 300-600 bp fragments using the Blue Pippin Prep System (Sage Science). Finally, fragments were amplified using a quantitative-PCR with 18 cycles (NEB Phusion high-fidelity polymerase, New England Biolabs), size-selected again for 300-600 bp fragments, and sequenced on an Illumina HiSeq 2500 system. The ngsComposer pipeline was used for fastq data filtering (Kuster et al., 2021). GBSapp, the corresponding bioinformatics pipeline, described previously (Wadl et al., 2018), was used for SNP/dosage calling and variant filtering. The GBSapp pipeline integrates original and third-party (samtools, picard, bcftools, GATK, NextGenMap, R-ggplot2, and R-AGHmatrix) tools to ensure implementation of variant calling best practices and reproducible results.

### Population genetics analyses

Several samples were excluded from the population genetic analyses due to discrepancies between repository data and root and foliar traits phenotyped and data collected for this study (Jackson et al., 2018; Jackson et al., 2019; Jackson et al., 2020). A total of 604 samples with 150,293 SNPs were generated after filtering and retained for analyses. Variant filtering was based on a minor allele frequency (MAF) threshold of 0.02 and read depth thresholds of 6, 25, and 45 for the 2x, 4x and 6x dosages, respectively. The SNP and sample missing rate were performed with a threshold of 0.3 and 0.3, respectively. Only the 6x dosage-based variants were used for downstream analyses. SNPs with a MAF of less than 0.05 were removed and linkage disequilibrium (LD) filtering was used to identify the most influential markers in the population to reduce computational costs. The ldsep v2.1.4 R Package (Gerard, 2021) was used to measure composite LD of SNPs through calculation of the squared Pearson’s correlation coefficient (r^2^) of SNPs on the same chromosome. Of the pairs of markers above the LD threshold of 0.1, the SNP with a lower MAF was eliminated from the dataset. MAF of the SNPs was estimated using PolyGene V1.4 (Huang et al., 2020) under the Disomic/Polysomic (RCS) inheritance model. MAF filtering and LD pruning resulted in elimination of 47,423 SNPs from the dataset.

PolyGene V1.4 was used to complete the following population genetic analyses on the reduced sample (n=604) and SNP (n=102,870) dataset. To assess population diversity, several parameters were calculated including polymorphic information criteria (PIC), effective number of alleles (Ae), allelic richness (Ar), Shannon’s Information Index (I), number of private alleles (NP), and F-statistics among populations. To account for geographical, ecological, and historical factors of gene flow and non-independence among individuals in this collection genetic differentiation was measured using Huang et al’s F_ST_ estimator (unpublished). Genetic distance was determined using Nei’s (1972) standard genetic distance (D_s_) (Nei, 1972). Finally, to examine population structure a Bayesian clustering analysis was performed using the admixture model with k runs from 1-10 and Markov Chain Monte Carlo (MCMC) parameters set to 5,000 burnin, 1,000 reps, and 10 runs. Population structure was further assessed using discriminant analysis of principal components (DAPC) in the adegenet v2.1.5 R package (Jombart and Ahmed, 2011). K-means clustering and Bayesian Information Criterion (BIC) were used to evaluate the appropriate number of clusters for further analyses. The final number of principal components was selected using alpha-score optimization, spline interpolation, and DAPC cross validation. The lowest mean squared error and highest mean success were used as criteria for selecting the optimal number of principal components to retain for DAPC analyses. In all, 150 principal components were retained for final iterations of DAPC.

### Determination of breeder germplasm subsets

Selection of breeder subsets began with generation of a full-autopolyploid kinship matrix of the 604 accessions genotyped with GBSpoly. Allele dosage data from the hexaploid collections were used to generate a full-autoploidy matrix (Slater et al., 2016). Resultant matrices were used to separate the accessions into 24, 48, 96, and 384 clusters. Complete hierarchical clustering was performed in R using the stats package and hclust function under the UPGMA clustering method.

We set out to minimize the inevitable loss of phenotypic diversity while also selecting manageable germplasm sets for use in future breeding/genetics studies. To accomplish this, accessions were manually selected with what we classified as extreme or rare phenotypes for each of 117 different sweetpotato descriptors (phenotypic traits) within the USDA, ARS, National Plant Germplasm System GRIN-Global database (https://npgsweb.ars-grin.gov/gringlobal/descriptors), with the goal of preserving diverse accessions within the breeder subsets. Of the initial 120 traits downloaded from GRIN-Global, 3 were discarded for not having enough data to be informative (“Longest Leaf Length (cm)”, “Width x Longest Length”, “Longest Length/Width”). We manually evaluated each of the other 117 phenotypes using custom R scripts, classifying each as either quantitative or categorical to identify individuals with rare phenotypes. The distribution of phenotypes for each trait was plotted as a histogram across all phenotyped individuals. For quantitative and semi-quantitatively measured traits, cutoff thresholds were either set at a 95% confidence interval (i.e. keeping accessions with the 5% highest and lowest measured phenotypes) for traits where the phenotypes were distributed in a visually bell-shaped manor, or alternatively were manually adjusted to retain the most extreme 10% of individuals for each trait. For categorical traits, we retained individual lines that had phenotypes that were present in less than 10% of individuals measured for a particular trait. In total, we preferentially retained 319 accessions which displayed an extreme phenotype in at least one trait. Because these 319 accessions did not encompass the entirety of the genetic diversity (only 231 of 384 clusters were represented), we also retained all the individual accessions (n=189) from the un-represented 384 clusters. This produced a final directed dataset of 508 accessions to select breeder subsets from (**Supplementary Table S1**). All R scripts and datasets are available on Ag Data commons (https://data.nal.usda.gov/dataset/slonecki-etal-scripts-and-datasets)

The phenotypic selections were used to augment the breeder subset selections made using the genotype data. If there were multiple lines within the same genetic cluster, we selected those that had rare phenotypes when available, otherwise all lines had the same chance of being selected for the breeder subset based on the genotypic data. By preferentially retaining individual accessions with extreme or rare phenotypes, we increased the likelihood of selecting the most phenotypically diverse breeder subset based on the data available in the GRIN-Global database. Criteria included total loss of phenotypes across all categorical traits, average reduction in measured phenotypic spread across all quantitatively and semi-quantitatively measured traits, and average deviation from the phenotypic mean across all quantitative and semi-quantitative traits. As expected, the breeder subsets selected from the reduced dataset had on average lower scores (less loss) for each of the meta criteria compared to those breeder subsets selected from the un-reduced dataset. Additionally, for each criterion the ‘best’ breeder subsets (i.e. having the least phenotypic diversity loss as measured by a specific criteria) were selected from the directed dataset. This confirmed that sampling from the directed dataset improved our chances of selecting more phenotypically diverse breeder subsets.

Using our directed dataset, we set out to select nested breeder subsets of 24, 48, 96, and 384 accessions. We started by iteratively selecting a 24-accession germplasm set based on the K=24 genomic clustering data, randomly selecting one accession from each cluster. For each selection we fixed the sweetpotato cultivars Beauregard (PI 566613), Porto Rico (PI 566646), Ruddy (PI 657999), and Tanzania (PI 595887). We performed 1,000 iterative selections and collected 6 different metadata criteria the were used to help pick the ‘best’ subset selection possible aimed at minimizing the loss of phenotypic diversity across all the traits from the GRIN-Global database. These metadata criteria included the total, average, and maximum observed loss of phenotypes across all categorical traits, as well as the average and maximum reduction in measured phenotypic spread across all quantitatively and semi-quantitatively measured traits and the average deviation from the phenotypic mean across all quantitative and semi-quantitative traits. After performing the iterative subset germplasm selections and aggregating all the metadata, we took a multi-criteria decision-making approach, identifying the breeder set selections that were in the lowest 21% quantile for all 6 criteria. We then chose the breeder set with the lowest (i.e. least loss) score for a majority of the six criteria. We repeated this process for selecting the 48, 96, and 384-accession breeder subsets, each time fixing the individuals from the previously selected breeder subset so that we developed 4 nested breeder subsets (**Supplementary Table S1**).

## Results

### Bioinformatic processing and SNP identification

The raw fastq files were generated by sequencing multiplexed libraries on an Illumina HiSeq2500 platform. The median Phred quality scores of the raw sequences ranged from 34 (first 5 of 6 bases in buffer sequence) to 38 (barcode region and genomic insert), with the last position (125-bp) having a median of 37 (**Supplementary Figure S1**). The minimum quality score of boxplot lower quartile and whiskers were the same as the median score along most of the reads. After demultiplexing and quality filtering, all the bases within the variable length barcode region (downstream of 6-bp buffer sequences that were trimmed off) and the genomic insert had high-quality median Phred scores of 38 or approximately 99.984% accuracy (**Supplementary Figure S2**). The minimum Phred score of the boxplot lower quartiles and whiskers were also 38 except for the last 2 base positions that have scores of 36-37 and 33-36, respectively.

After aligning the demultiplexed and filtered reads to the two ancestral reference sub-genomes (Sweetpotato Genomics Resource at Michigan State University, 2018), on average, 96.6% of the raw reads mapped to both reference genomes (*I. trifida* and *I. triloba*). There tended to be a higher proportion of reads specific to the *I. triloba* subgenome than those reads specific to the *I. trifida* subgenome. Read depth distribution across all samples were fairly uniform (**Supplementary Figure S3**). The median and mean read depth across the samples were 25 and 66, respectively. After variant calling, read depth filtering was performed for each data point independently and. minor allele frequency (MAF) filtering was performed with MAF >= 0.02 (median and mean MAF are 0.12 and 0.17, respectively, **Supplementary Figure S4**). The marker density for the 2x, 4x and 6x dosage-based genotypes were 85,729, 207,629, and 150,293, respectively (**Supplementary Table S2**). Only the 6x-dose genotypes were used for subsequent analysis.

### Genetic diversity and population structure of sweetpotato collection

After exclusion of samples with missing or incorrectly recorded data, a total of 604 sweetpotato accessions from 7 defined geographical regions were retained for population genetics analysis (**Table 1** and **Supplementary Table S1**). The GBSpoly pipeline resulted in a total of 150,293 SNPs genotyped for each of these accessions. To eliminate nonrandom association of alleles and prevent skewed downstream analyses, SNPs were pruned with LD threshold of 0.1 and SNPs with a MAF < 0.05 were eliminated. In total, 102,870 SNPs were retained for further analyses.

**Figure 1:**
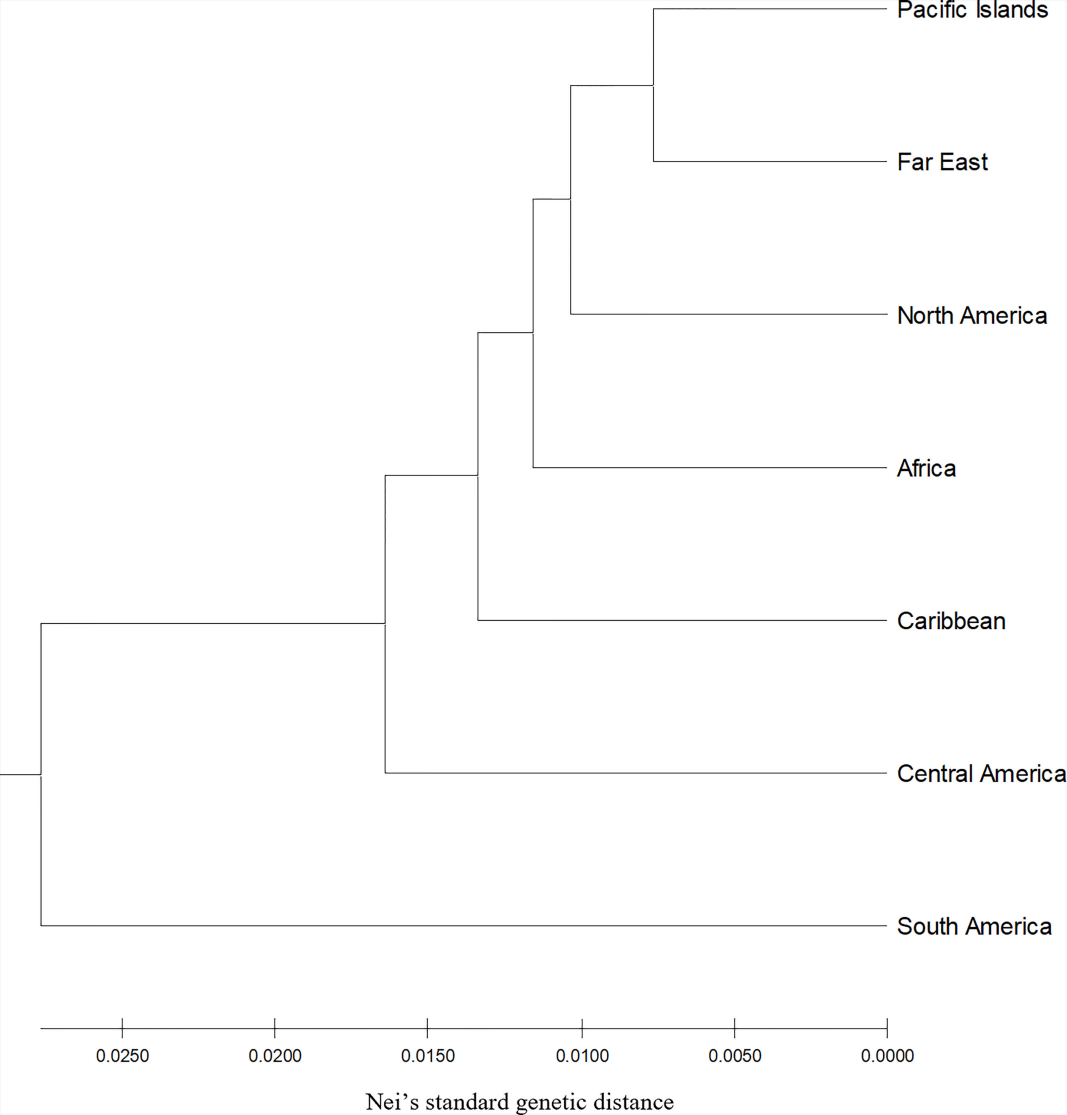
Hierarchical clustering unweighted pair group method with arithmetic mean by geographic region using Nei’s standard genetic distance of 604 sweetpotato (*Ipomoea batatas*) accessions.

The number of distinct alleles (k) at a locus averaged 5.503 across all accessions and ranged from 3.664 (North America) to 3.906 (Central America) (**Table 1**). Polymorphic information content (PIC) values averaged 0.353 across all accessions and ranged from 0.321 (North America) to 0.359 (Central America) within region accessions, whereas Shannon’s information index (I) averaged 0.797 across all accessions and ranged from 0.699 (North America) to 0.775 (Central America) among region accessions. The effective number of alleles (Ae) averaged 2.076 across all accessions and ranged from 1.966 (South America) to 2.090 (Caribbean) within region accessions, while allele richness (Ar) averaged 6.226 across all accessions and ranged from 4.431 (South America) to 4.970 (Central America) within geographic regions. Finally, the number of private alleles (NP) averaged 5.503 among the entire population and ranged from 0.052 (North America) to 0.186 (Central America) within populations. Across all geographic regions, genetic differentiation (F_ST_) averaged 0.051, while South American accessions were vastly different compared to all other geographic regions with all F_ST_ values > 0.077 (**Table 2**). All other F_ST_ pairwise comparisons not involving South America were ≤ 0.051, while South America and Central America had the maximum F_ST_ value of 0.088. The genetic distance (Nei’s standard genetic distance) (Nei, 1972) between all accessions ranged from 0.13 to 0.67 with a mean of 0.50. When comparing geographic regions, genetic distance ranged from 0.015 to 0.060 with a mean of 0.034. South America appeared to be the most genetically distant from all regions with an average distance of 0.054. Unweighted pair group method with arithmetic mean (UPGMA) hierarchical clustering of genetic distance by region and accession allowed for easier visualization of genetic distances (**Figure 1**).

**Table 2:** Pairwise genetic differentiation (F_ST_ Huang unpub estimator – PolyGene V1.4) between geographical regions of 604 sweetpotato (*Ipomoea batatas*) accessions.

	Africa	Caribbean	Central America	Far East	North America	Pacific Islands	South America
Africa	0.000						
Caribbean	0.039	0.000					
Central America	0.051	0.049	0.000				
Far East	0.033	0.039	0.038	0.000			
North America	0.034	0.034	0.049	0.030	0.000		
Pacific Islands	0.030	0.034	0.039	0.020	0.031	0.000	
South America	0.085	0.084	0.088	0.071	0.085	0.077	0.000

F_ST_ among all populations was 0.051.

**Figure 2:**
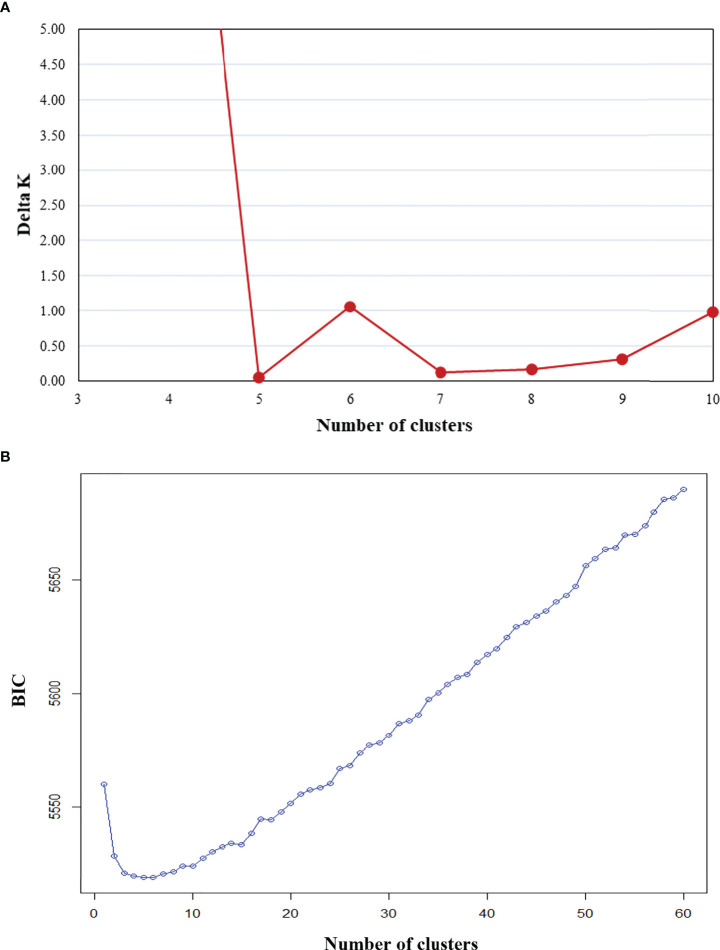
Clustering of 604 sweetpotato (*Ipomoea batatas*) accessions via two methods: **(A)** K-means clustering and **(B)** Bayesian clustering.

**Figure 3:**
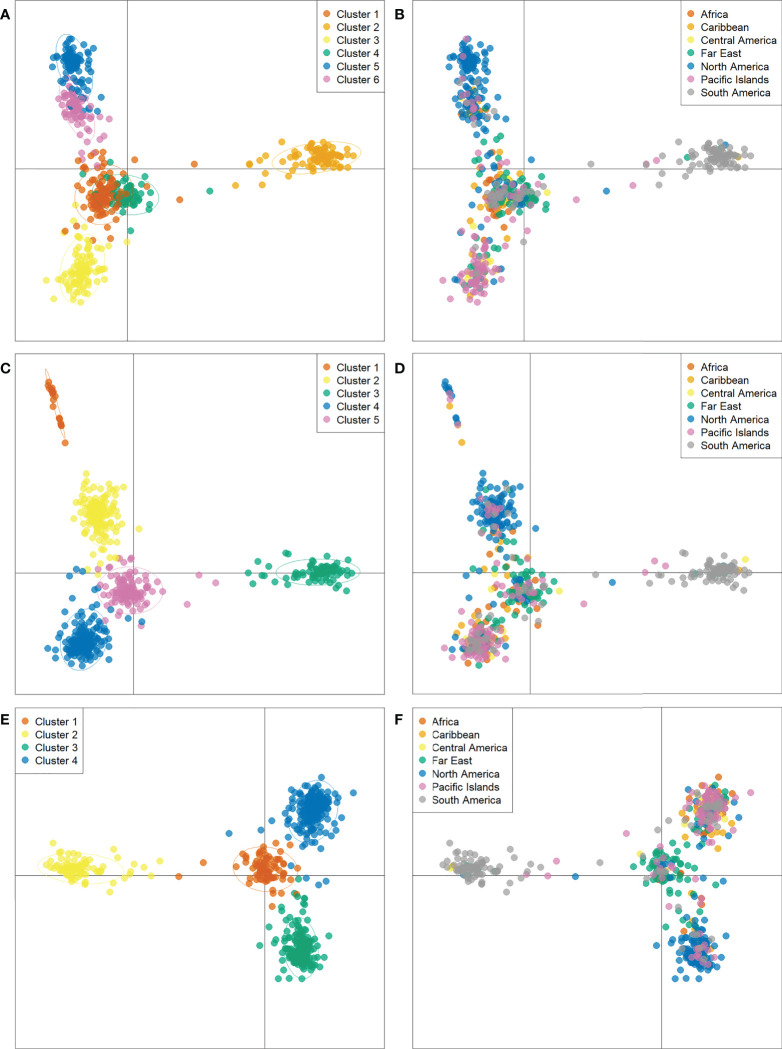
Discriminant analysis of principal components (DAPC) of 604 sweetpotato (*Ipomoea batatas*) accessions scatterplots representing K=6 **(A, B)**, K=5 **(C, D)**, and K=4 **(E, F)** labeled by cluster assignment and country of origin respectively.

While determining assignment of accessions to a specific population cluster is an imprecise practice, it can still provide valuable information regarding genetic diversity and help paint a picture of historical genetic movement between populations. Here, potential population groupings were evaluated using three approaches. First, within PolyGene V1.4, Bayesian clustering was performed using the admixture model with k runs from 1-10 and Markov Chain Monte Carlo (MCMC) parameters set to 5,000 Burnin, 1,000 reps, and 10 runs. Delta K values were calculated from ln P(D) and peak delta K was observed at K=6 (**Figure 2A**). Second, K-means clustering and Bayesian Information Criterion (BIC) were used to predict the number of population clusters with 600 retained principal components. The lowest BIC values were observed at K=4-6 with K=5 as the minimum (**Figure 2B**). As the number of clusters increased beyond K=6, BIC inflected upward and maintained a consistent trajectory higher. A local valley, as observed in this K-means-BIC analysis, is to be expected when the population is clustering into distinct groups. Finally, discriminant analysis of principal components (DAPC) was used to visualize accession clustering by geographical region. Groupings of 4, 5, and 6 were chosen based on Bayesian clustering, and K-means clustering (**Figure 2**). Among the first two principal components in all groupings, accessions from South America clustered most distinctly. Clustering of 5 and 6 groupings revealed slight separation of accessions from North America into 2 distinct groups when compared to clustering with 4 groupings (**Figure 3**). Furthermore, while not as clear as other divisions, K=6 clustering suggested that cluster 4, which is largely composed of Far East accessions, may be drifting away from cluster 1, which is a mix of all geographic regions. DAPC cluster (K=6) assignment by country and principal coordinate analysis of the same dataset supported similar findings (**Table 3** and **Supplementary Figure S5**).

**Table 3:** Discriminant analysis of principal components (DAPC) cluster assignment by region of 604 sweetpotato (*Ipomoea batatas*) accessions.

Region	DAPC cluster (no. of accessions)					
	**1**	**2**	**3**	**4**	**5**	**6**
Total	150	96	107	64	93	94
Africa	38	0	1	0	2	6
Caribbean	19	1	5	0	1	5
Central America	15	1	14	1	0	2
Far East	28	4	17	43	4	8
North America	13	1	8	6	80	60
Pacific Islands	17	4	59	9	3	10
South America	20	85	3	5	3	3

**Figure 4:**
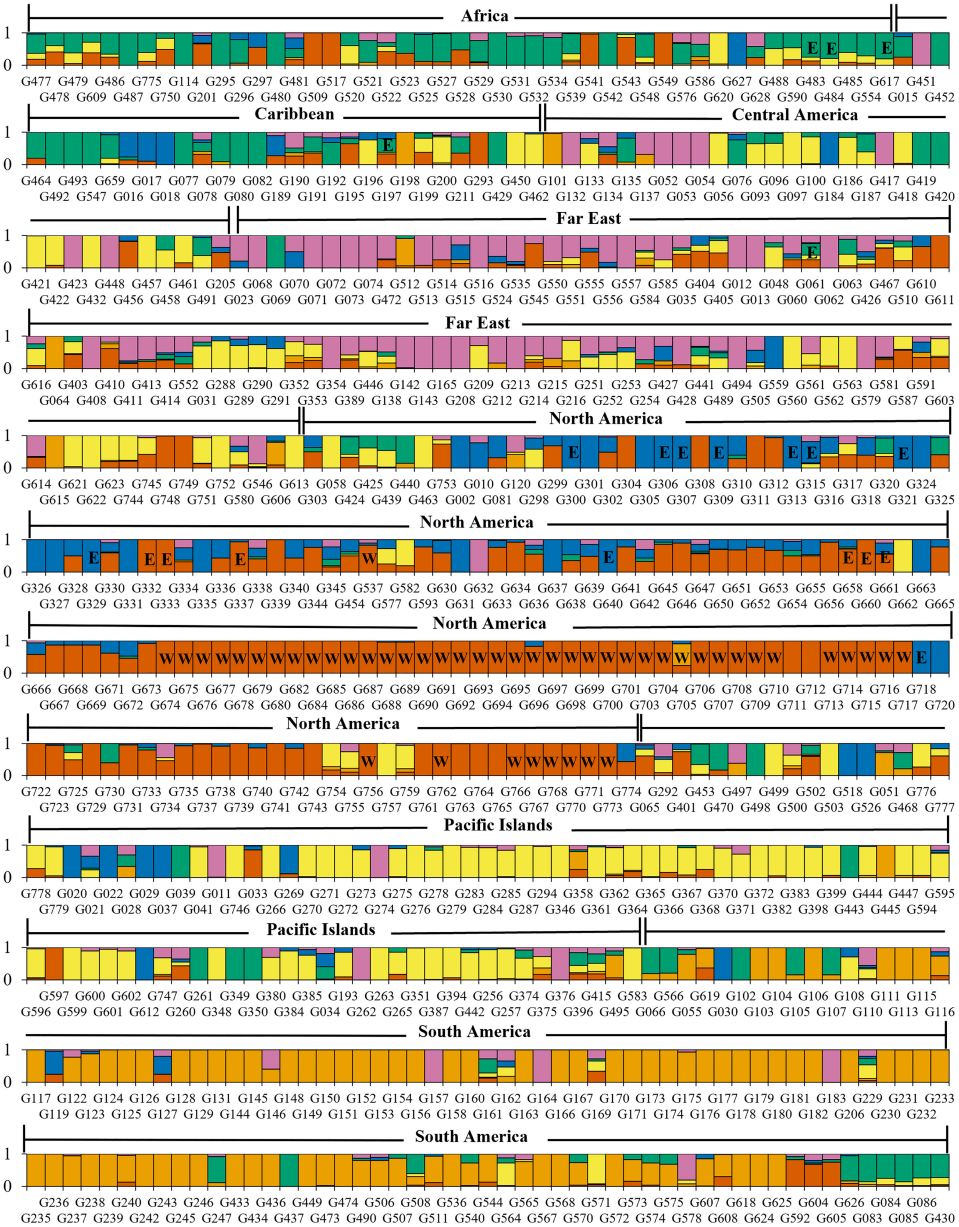
Barplot of Bayesian clustering assignments of 604 sweetpotato (*Ipomoea batatas*) accessions for K=6. Assignment probabilities are shown for the six genetic groups identified where C1 is red, C2 is orange, C3 is yellow, C4 is green, C5 is blue, and C6 is pink. Accession bars labeled “E” have been identified as having *Meloidogyne enterolobii* resistance and accession bars labeled “W” are members of the W-lines.

Filtering inferred ancestry with a q-value threshold of 0.65 of the Bayesian clustering revealed that 74% of accessions were assigned to one of K=4 clusters, 77% of accessions were assigned to one of K=5 clusters, and 76% of accessions were assigned to one of K=6 clusters. When analyzing Bayesian and DAPC clustering together, K=4 appeared to capture a large degree of the genetic diversity observed among geographic regions, but failed to distinguish between more recent, localized diverging gene pools. Meanwhile, clustering at K=5 captured potential further discrimination between subgroups but did not distinctly separate any geographical groups. With a similar degree of admixture observed and greater division of North American and Far East accessions, K=6 may present a more accurate depiction of sweetpotato germplasm gene pools largely corresponding to crop improvement program selection over the past century. Review of K=6 revealed Bayesian clusters of 114, 94, 102, 58, 40, 54 accessions with 142 accessions not assigned due to admixed ancestry (**Figure 4** and **Supplementary Figure S6**). Cluster 1 (n=114), primarily consisted of accessions from North America (n=95); cluster 2 (n=94) was composed of accessions almost entirely from South America (n=84); cluster 3 (n=102) was mostly from the Pacific Islands (n=60), cluster 4 (n=58) was a mix of Africa (n=20), Central America (n=12), and South America (n=13); cluster5 (n=40) was largely from North America (n=24); and cluster 6 (n=54) was principally composed of accessions from the Far East (n=36).

### Breeder subset selection

Starting with the 604 genotyped accessions, we manually selected nested breeder subsets of 24, 48, 96, and 384 to minimize the loss of genetic and phenotypic diversity from the GRIN-Global database. We preferentially retained accessions that were reported to have ‘rare’ phenotypes (present in less than 10% of phenotyped lines for a specific trait) for one or more of 117 traits that were available from the GRIN-Global database. This created a smaller directed sampling set of 508 accessions that still retained accessions from all 384 genetic clusters from the original data set. From this directed set, we iteratively sampled 1,000 potential breeder subsets at each size (a breeder set = one accession selected from each genetic cluster at K=24, 48, 96, or 384) and compared each potential breeder set back to the complete 604 accession dataset to assess the loss of phenotypic diversity. We used a multi-criteria decision-making approach to select a breeder subset with the lowest (i.e. least loss) score across six phenotypic loss criteria. For the 48 categorical traits we assessed the total, average, and maximum loss of phenotypes, and for the other 68 quantitatively or semi-quantitatively measured traits we assessed the average deviation from the phenotypic mean for each trait as well as the average and maximum reduction in measured phenotypic spread. All 4 selected breeder subsets ranked within the top 10-21% across all six criteria when compared against the other randomly selected sets of the same size, displaying less phenotypic loss then would be expected by random selections based on the genetic clustering data alone (**Supplementary Figure S7**).

**Figure 5:**
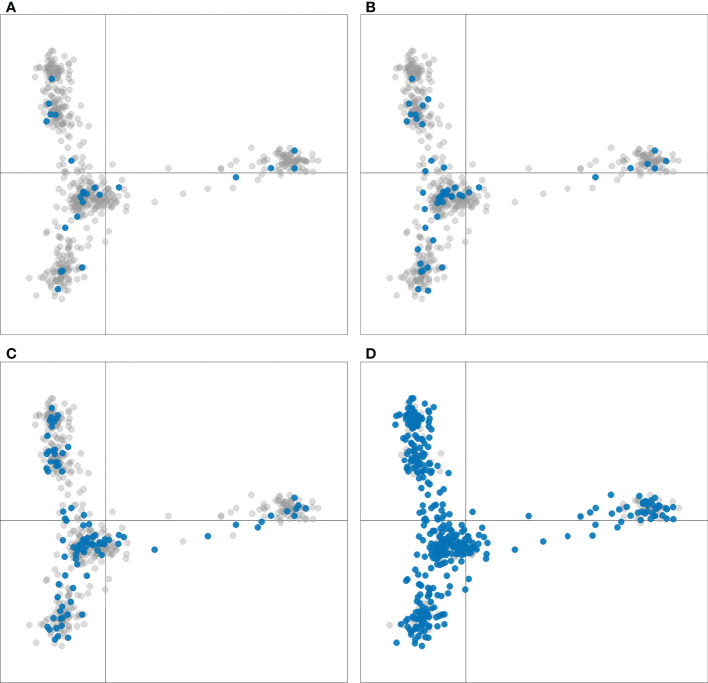
Discriminant analysis of principal components (DAPC) of 604 sweetpotato (*Ipomoea batatas*) accessions scatterplots for K=6. Accessions included in the breeder subsets of **(A)** 24, **(B)** 48, **(C)** 96, and **(D)** 384 are colored blue, all other accessions are colored gray.

The number of accessions in each breeder subset from the geographical regions discussed (**Table 1** and **Supplementary Table S1**) is an indication of preservation of geographic and genotypic diversity among the breeder subsets as well. Distinction of the breeder subsets within the six cluster DAPC demonstrated homogenous breeder subset selection across all clusters and further supports the preservation of genetic diversity among these sets (**Figure 5**).

### Linkage disequilibrium decay

Linkage disequilibrium (LD) decay is a vital tool for determining the resolution of association mapping of diverse germplasms (Vos et al., 2017). Using the ldsep R package, we estimated LD of markers on the same chromosome and used generalized additive model (GAM) smoothing to determine the level of LD decay. At thresholds of 0.2 and 0.1 (r^2^), LD decayed between 0 and 0.7 kb (**Supplementary Figure S8**). Accordingly, LD blocks for the 604 sweetpotato accessions appear small enough for genome wide association mapping.

## Discussion

Due to its nutritional and caloric value, the sweetpotato is a critically important crop worldwide. Likely because of public awareness of its health benefits, sweetpotato has become increasingly more popular in the United States since 2000 (Economic Research Service, United States Department Of Agriculture, 2016). This growth in popularity has boosted the need for greater characterization and diversity selection within the USDA sweetpotato germplasm collection. This study genotyped and characterized 604 accessions from the PGRCU sweetpotato germplasm collection. Genetic diversity and population structure were assessed *via* 102,870 SNPs. Additionally, K-means clustering and specific selection were used to create breeder germplasm subsets of 24, 48, 96, and 384 accessions to facilitate phenotypic characterization of genetically diverse germplasm within the USDA germplasm collection.

Analyses of sweetpotato germplasm collections with GBS data have recently been conducted. Germplasm from 2 USDA collections (n=417) found that accessions were clustered into 4 genetic groups (Wadl et al., 2018), whereas an analysis of accessions primarily from China (n=197) found that 3 genetic groups best describe the material in that collection (Su et al., 2017). More recently, the International Center of Potato (CIP) genotyped 45% of the collection (n=5,979) using 20 simple sequence repeat markers and concluded 4 genetic clusters best describe the collection (Anglin et al., 2021). While it is difficult to discern true population structure and clusters, we found that historical and experimental evidence from this study suggests that K=6 may better encapsulate sweetpotato population structure of the PGRCU collection. Clustering at K=5 is an alternative option, but one that will likely leave out several diverging population clusters observed with larger datasets.

Our DAPC and Bayesian clustering approach highlighted divergence within the North American sweetpotato accessions; separating this population into two distinct clusters. One cluster primarily consisted of germplasm derived from the USDA, ARS, United States Vegetable Laboratory (USVL) and the other cluster consisted of germplasm from other United States public breeding programs. As suggested previously (Wadl et al., 2018), this divide is likely due to key selection differences within the North American germplasm, specifically, the introgression of the cultivar Porto Rico (individual G329, **Figure 4**, PI 566646) into the United States sweetpotato industry in 1906 and the USVL sweetpotato breeding program development of mass-selection populations (Harmon et al., 1970). Bayesian clustering of the PGRCU collection (**Figure 4**) shows a significant divide between W-lines (marked ‘W’) and material with genetic similarities to ‘Porto Rico’, including ‘Carver’ (G306; PI 566618), ‘Jewel’ (G321; PI 566638), and ‘Hayman White’ (G640; PI 634380), among others, further supporting the DAPC analysis. The USVL mass-selection populations, from which the W-lines and other cultivars were publicly released into the PGRCU collection, focused on selection of material for multiple insect, nematode, and disease resistance as well as other desirable production and market qualities (Jones et al., 1991). This heavy selection pressure likely created a divide in the gene pool between breeding programs within North America. Amongst the collection, several suspected duplicate accessions were identified based on genotype data and common names [PI 606268 and 612696 (Helena), PI 531147 and 573295 (Tainung 57), PI 595888 and 633966 (Wagabolige), and PI 599385 and 606278 (IITA-TIS 9101)] and served as controls to substantiate DAPC and Bayesian clustering.

Among the entire PGRCU collection, diversity and clustering analysis demonstrated the substantial genetic divide between accessions from South America and all other geographic regions. Not only were regional pairwise genetic differentiation and distance values for South America higher than average (F_ST_ ≥ 0.71, genetic distance ≥ 0.48), Bayesian clustering exhibited minor admixture with other geographic regions. Upon population structure analysis of the CIP collection, Anglin et al. suggested that accessions from Peru had been genetically isolated over time (Anglin et al., 2021). Of the 85 accessions from South America clustered in DAPC cluster 2 (**Figure 3A** and **Table 3**), 84 were from Peru, further confirming this idea. While there is a high level of admixture in the Far East and Africa and many genetic similarities to Central America and Caribbean accessions, (**Supplementary Figure S6**), DAPC clustering suggests that accessions from the Far East and Africa may be converging into unique gene pools. Explanations for such convergence are lacking, however, much of the overlap between African, Central American, and Caribbean gene pools may have been facilitated by the colonial slave trade (Loebenstein and Thottappilly, 2009).

Among the goals of this diversity and population analysis was to select representative samples or breeder subsets as proposed by Franco-Duran et al. (2019) through the development of custom R scripts that ensured selection of a genotypically and phenotypically diverse collection of germplasm for future breeding and genetic studies, such as GWAS. Maintaining the phenotypic and genotypic diversity of a germplasm collection can quickly strain resources and become impractical with increasing numbers of accessions, especially in the case of a clonally propagated crop such as sweetpotato where maintaining accessions can be very expensive. Another confounding factor is the sparse passport and characterization data available for the PGRCU collection. Highlighting the breeder subsets within the DAPC analysis (**Figure 5**) demonstrated homogeneity of selected accession across the PGRCU collection clusters. Furthermore, the freely available R scripts developed in this study in combination with available genotype and phenotype data can be used by plant breeders and molecular geneticists to select their own specific sweetpotato breeder subsets based on specific programmatic needs. Additionally, these R scripts could be modified and utilized as a tool for developing specific breeder subsets for other crops.

An emerging concern within the United States is the highly virulent guava root-knot nematode (*Meloidogyne enterolobii*), which causes significant damage to a wide range of crops and has become a serious problem in the US sweetpotato industry (Rutter et al., 2021). Although sources of resistance to this introduced nematode species have been identified (Rutter et al., 2021; Schwarz et al., 2021), the most popular cultivars grown in the United States are highly susceptible. Identification and introduction of cultivars with resistance will be crucial in efforts to control this emerging pest of sweetpotato. Based on accessions with known *M. enterolobii* resistance (**Figure 4**, marked “E”) we speculate that resistance may have arisen from different backgrounds. Fortunately, *M. enterolobii* resistance has been found in multiple cultivars [Apache (G300; PI 566611), Carver (G306; PI 566618), Centennial (G307; PI 566619), Georgia Red (G313; PI 566628), Hayman White (G640; PI 634380), Heartogold (G315; PI 566631), Jewel (G321; PI 566638), Pelican Processor (G658; PI 634398), Porto Rico (G329; PI 566646), Red Resisto (G660; PI 634400), Regal (G332; PI 566650), Resisto (G333; PI 566651), and Southern Delite (G337; PI 566655)] released from the prevalent and historical North American breeding programs and is from two distinct genetic backgrounds (**Figure 4**). Resistance is also present in ‘Tanzania’ (G483; PI 595887) and ‘Wagabolige’ [G484; PI 595888 (**Figure 4**)] that were released by the Ugandan breeding program in 1995 (Mwanga et al., 2001). The Bayesian and DAPC cluster analyses indicate that resistance is present in four different genetic backgrounds and the *M. enterolobii* resistant ‘Mojave’ consists of all four [**Figure 4** (G197; PI 538351)]. Additional phenotyping studies with the 384 breeder subset will help identify markers associated with *M. enterolobii* resistance and speed up breeding more diverse and resilient germplasm.

Development and maintenance of diverse and robust germplasm is crucial to ensuring food security worldwide, particularly for sweetpotato as it is an important food staple in the much of the developing world where malnutrition persists. The wealth of knowledge gained through genotyping and phenotyping studies allows for effective breeding of pest, disease, and environmental stress resistance/tolerance traits to support germplasm diversification and resource availability. The genetic characterization of the USDA, ARS, PGRCU germplasm collection and the tools provided herein to develop the breeder subsets in this study provide the genetic foundation for more phenotyping studies aimed at linking genotype with phenotype. Stemming from this future research, markers associated with well phenotyped germplasm will impact our understanding of genetic diversity, population structure, and provide a framework for development of resilient United States sweetpotato germplasm.

## Data availability statement

The datasets presented in this study can be found in online repositories. The names of the repository/repositories and accession number(s) can be found below: https://www.ncbi.nlm.nih.gov/, PRJNA880973, https://github.com/William-Rutter-USDA-ARS/Sweetpotato_Core_set_Slonecki_etal.git.

## Author contributions

TS and WR: performed research, analyzed data, wrote the manuscript. BO: designed research, performed research, analyzed data, and wrote the manuscript. GY: designed research, wrote the manuscript. DJ: performed research, wrote the manuscript. PW: designed research, performed research, analyzed data, and wrote the manuscript. All authors contributed to the article and approved the submitted version.
