# Supplementary material for: Genetic diversity, population structure, and selection of breeder germplasm subsets from the USDA sweetpotato (Ipomoea batatas) collection
Source: Front Plant Sci. 2023 Feb 2;13:1022555. doi: 10.3389/fpls.2022.1022555 (PMC9932972; doi:10.3389/fpls.2022.1022555)
Supplement: Supplementary file 1 [file DataSheet_1.docx]

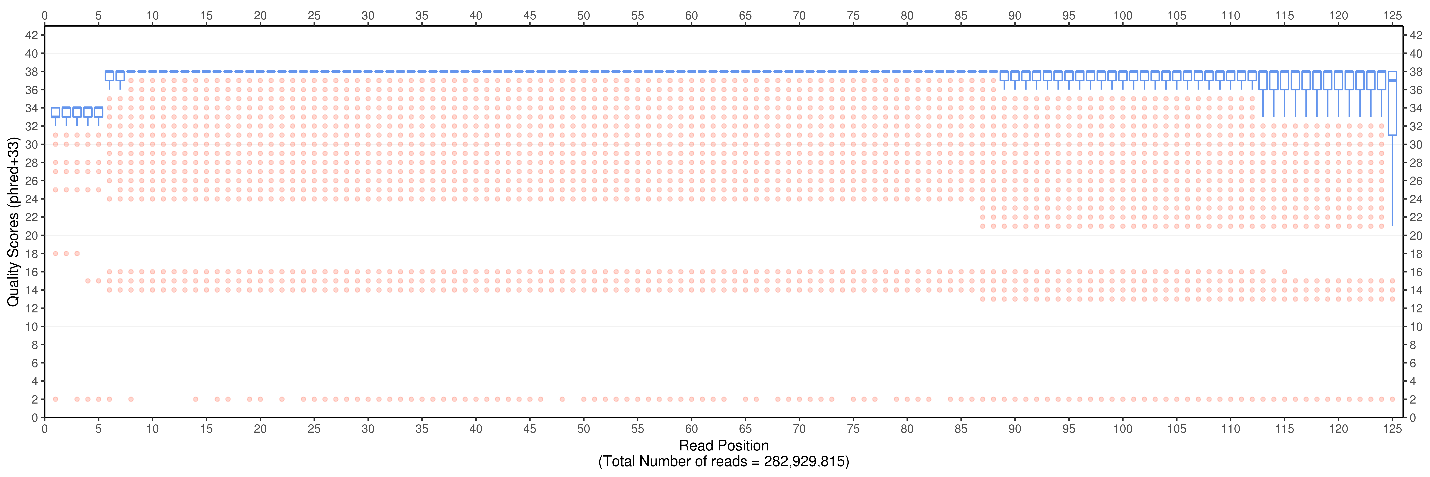
**Figure S1.** Phred quality scores of raw sequences generated by sequencing multiplexed libraries on an Illumina HiSeq2500.


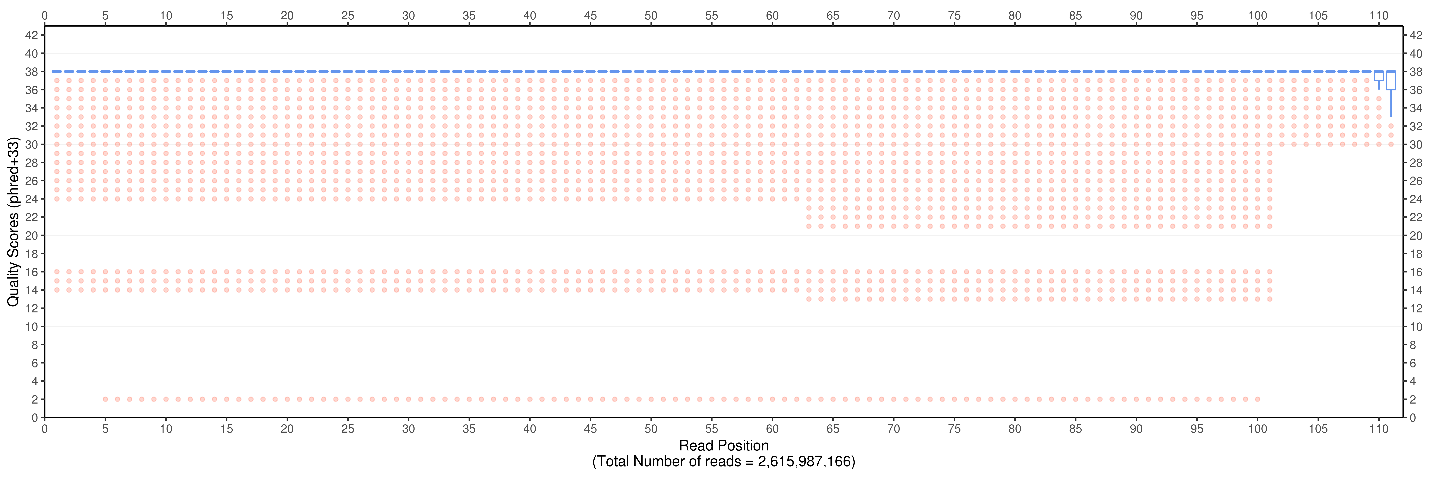


**Figure S2.** Phred quality scores of demultiplexed and quality filtered sequences generated by sequencing multiplexed libraries on an Illumina HiSeq2500.


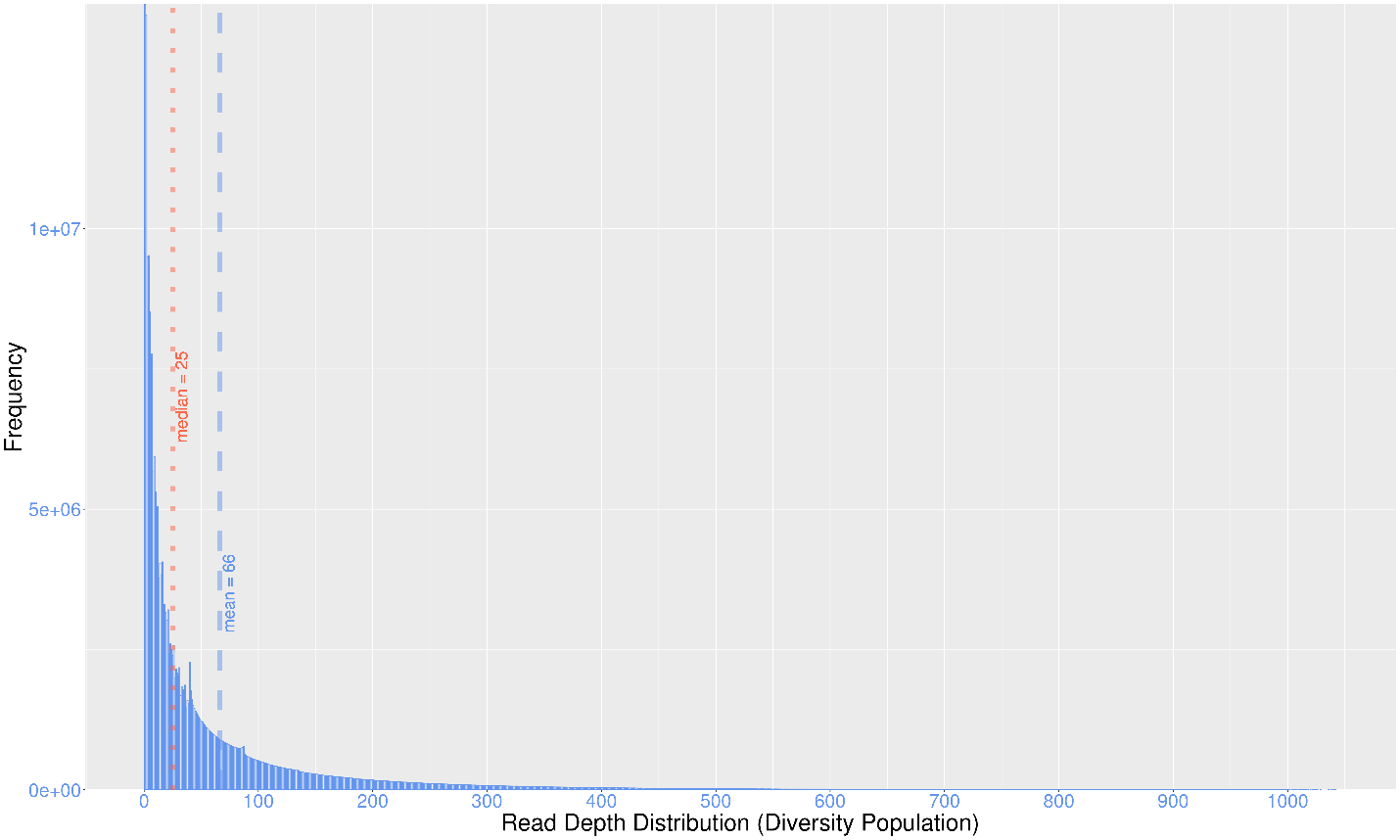


**Figure S3.** Read depth distribution of the 6x diversity population. Median = 25 and mean = 66.


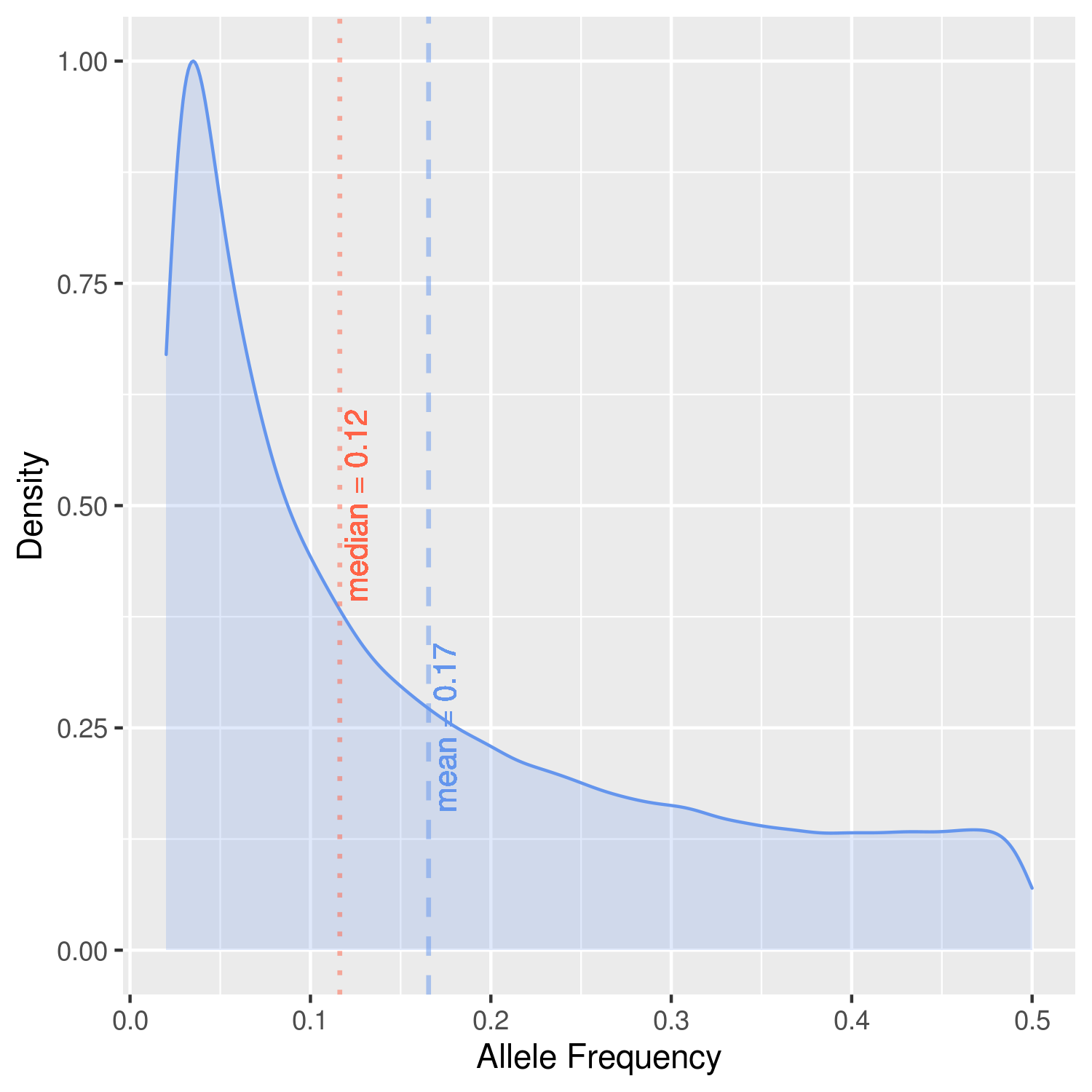


**Figure S4.** Distribution of minor allele frequency (MAF) among 6x diversity population. Median = 0.12 and mean = 0.17.


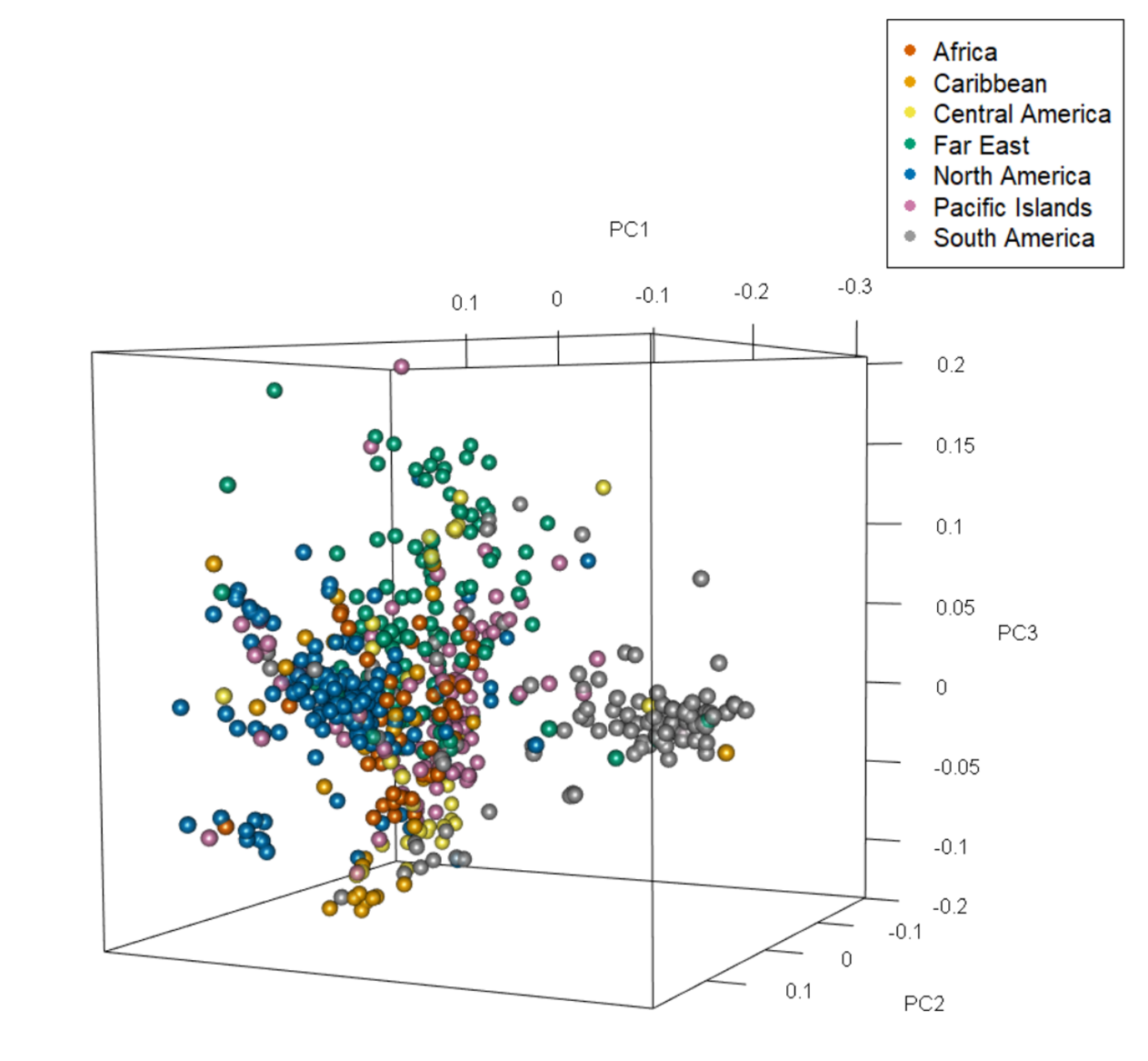


**Figure S5.** Principal coordinate analysis (PCoA) of 604 sweetpotato (*Ipomoea batatas*) accessions scatterplot of first three principal components. Accessions colored by geographic regions include Africa (red), Caribbean (orange), Central America (yellow), Far East (green), North America (blue), Pacific Islands (pink), and South America (gray).


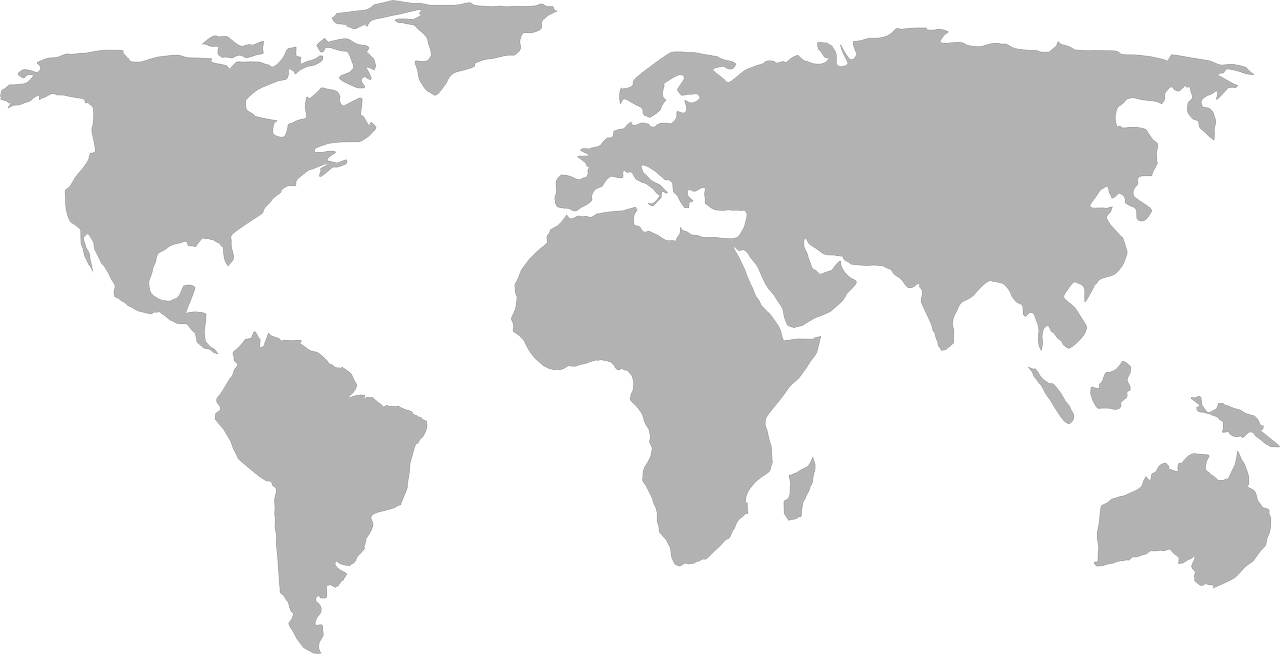

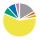

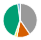

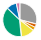

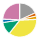

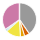

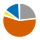

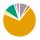


**Africa (n = 47)**

**Far East (n = 104)**

**Pacific Islands (n = 102)**

**North America (n = 168)**

**Central America (n = 33)**

**South America (n = 119)**

**Caribbean (n = 31)**

**Figure S6.** Map of Bayesian clustering assignments of 604 sweetpotato (*Ipomoea batatas*) accessions. Regional pie charts represent proportional cluster assignments for the six genetic groups identified where C1 is red, C2 is orange, C3 is yellow, C4 is green, C5 is blue, C6 is pink, and admixed is gray. A q-value threshold of 0.65 was used to separate admixed accessions from the other cluster assignments.


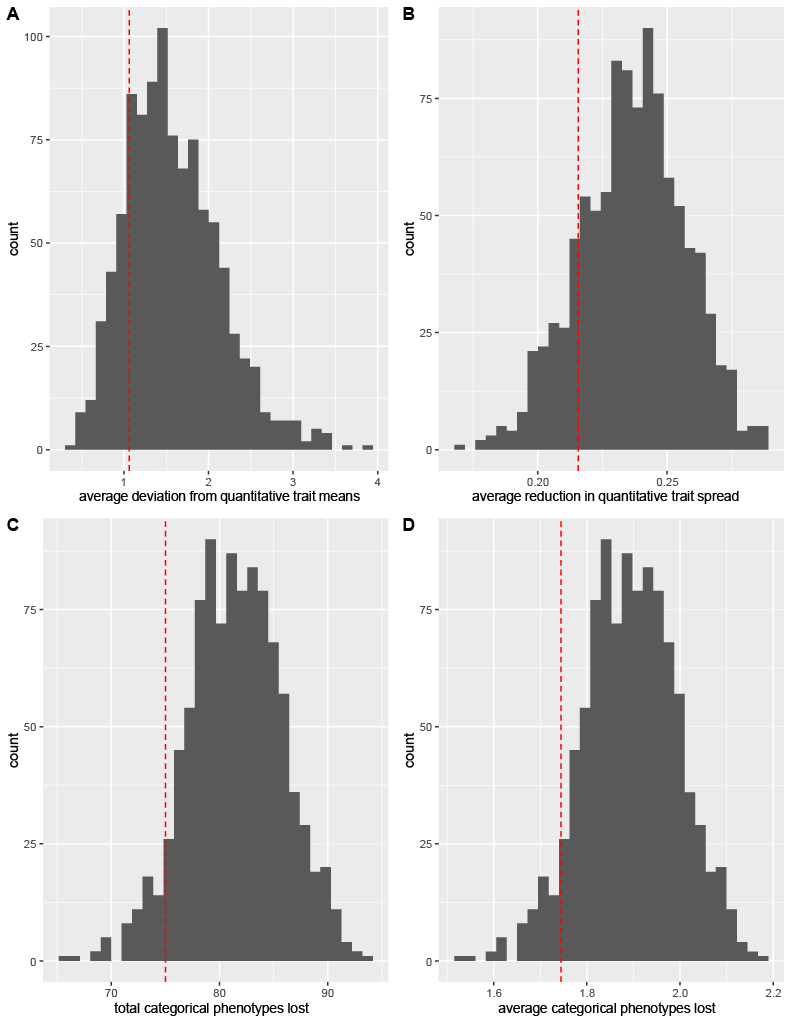


**Figure S7.** Histograms depicting the phenotypic metadata collected from 1 000 possible 96-accession core sets generated. The vertical red line indicates the data from our selected 96-accession core set, which was in the top 10% across all criteria when compared to the other possible core sets of the same size. A&B) depict the average deviation from the mean and average reduction in phenotypic spread across 68 quantitatively measured traits. C&D) depict the total and average number of phenotypes lost across 48 categorical traits.


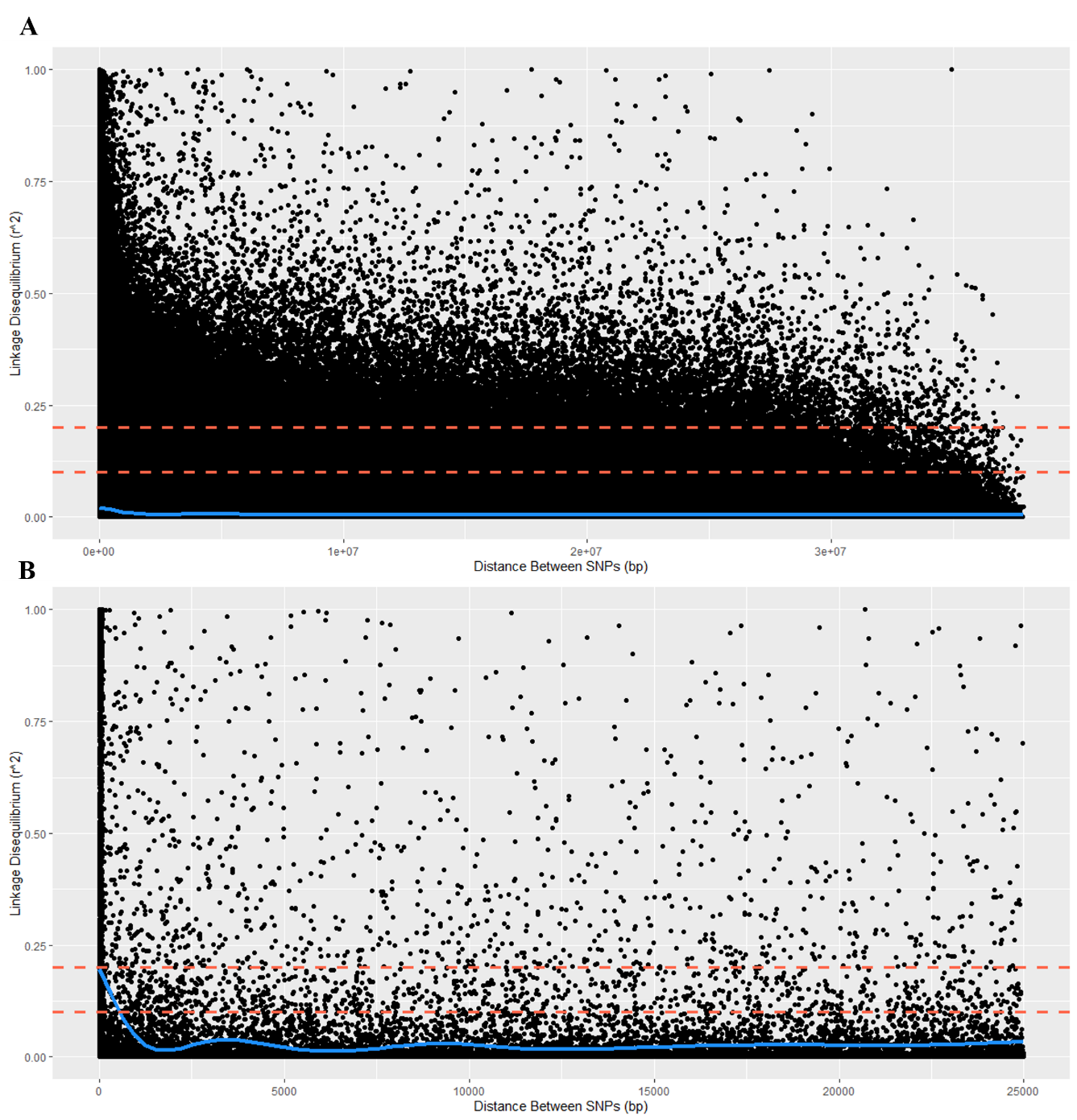


**Figure S8.** Scatterplots of sweetpotato germplasm (604 accessions) SNP pair linkage disequilibrium (r^2^) against respective SNP base pair (bp) intervals. Red dashed horizontal lines represent LD thresholds of 0.1 and 0.2. A generalized additive model (gam) was used to illustrate the distribution of all points (blue curve). Plots of (A) maximum bp and (B) limited 25 000 bp intervals were generated from the same dataset.

**Supplementary table captions – included as Excel documents**

**Table S1.** Data table detailing accession details, clustering, and core selection sets for 604 sweetpotato accessions from the USDA PGRCU germplasm.

**Table S2.** Data table detailing the number of SNPs and samples eliminated by missing variant and sample missing rate.
