# Supplementary material for: Genetic diversity, population structure, and selection of breeder germplasm subsets from the USDA sweetpotato (Ipomoea batatas) collection
Source: Front Plant Sci. 2023 Feb 2;13:1022555. doi: 10.3389/fpls.2022.1022555 (PMC9932972; doi:10.3389/fpls.2022.1022555)
Supplement: Supplementary file 4 [file Table_3.docx]

Standard Illumina adapters for the amplification of the library.

Illumina-P5 5`-AATGATACGGCGACCACCGAGATCTACACTCTTTCCCTACACGACG-3`

Illumina-P7 5`-CAAGCAGAAGACGGCATACGAGATGTGACTGGAGTTCAGACGTGTGC-3`
